# Supplementary material for: Near index matching enables solid diffractive optical element fabrication via additive manufacturing
Source: Light Sci Appl. 2023 Sep 12;12:222. doi: 10.1038/s41377-023-01277-1 (PMC10495398; doi:10.1038/s41377-023-01277-1)
Supplement: Supplementary file 1 — Supplementary Information [file 41377_2023_1277_MOESM1_ESM.docx]

Supplementary Information for

**Near index matching enables solid diffractive optical element fabrication via additive manufacturing**

Reut Orange Kedem^1,2^, Nadav Opatovski^1,2^, Dafei Xiao^1,2^, Boris Ferdman^1,2^, Onit Alalouf^2,3^, Sushanta Kumar Pal^4^, Ziyun Wang^5,6^, Henrik von der Emde^7^, Michael Weber^7^, Steffen J. Sahl^7^, Aleks Ponjavic^5,6^, Ady Arie^4^, Stefan W. Hell^7,8^, Yoav Shechtman^1,2,3,†^

^1^Russell Berrie Nanotechnology Institute, Technion—Israel Institute of Technology, Haifa, Israel
^2^Lorry Lokey Interdisciplinary Center for Life Sciences and Engineering, Technion—Israel Institute of Technology, Haifa, Israel
^3^Department of Biomedical Engineering, Technion—Israel Institute of Technology, Haifa, Israel
^4^School of Electrical Engineering Fleischman Faculty of Engineering, Tel Aviv University, Tel Aviv 69978, Israel
^5^School of Physics and Astronomy, University of Leeds, Leeds, UK
^6^School of Food Science and Nutrition, University of Leeds, Leeds, UK
^7^Department of NanoBiophotonics, Max Planck Institute for Multidisciplinary Sciences, Göttingen, Germany
^8^Department of Optical Nanoscopy, Max Planck Institute for Medical Research, Heidelberg, Germany

^†^ Corresponding author: [yoavsh@technion.ac.il](mailto:yoavsh@technion.ac.il)

**Table of Contents**

[Note 1: High order SPP Scan 2](#_Toc139476217)

[Note 2: Surface Roughness 4](#_Toc139476218)

[Note 3: Dispersion 7](#_Toc139476219)

[Note 4: Stability measurement 9](#_Toc139476220)

[Note 5: Performance comparison 9](#_Toc139476221)

[Note 6: Staircase effect 11](#_Toc139476222)

[Note 7: Choosing 3D printing technology and materials 12](#_Toc139476223)

[Note 8: Material homogeneity 13](#_Toc139476224)

[Note 9: Photon efficiency 15](#_Toc139476225)

[Note 10: Experimental PAINT performance comparison 16](#_Toc139476226)

[Note 11: Movie details 17](#_Toc139476227)

­

a

b


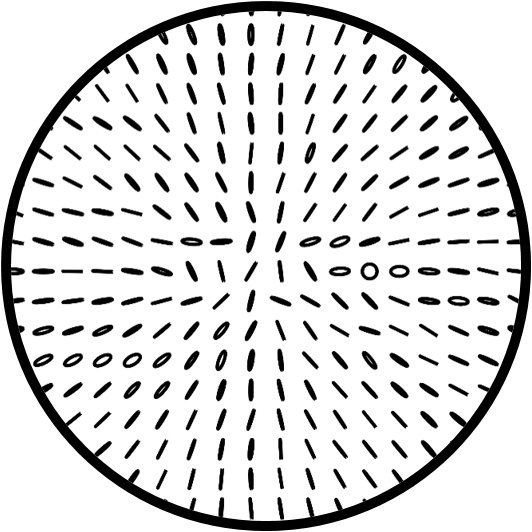

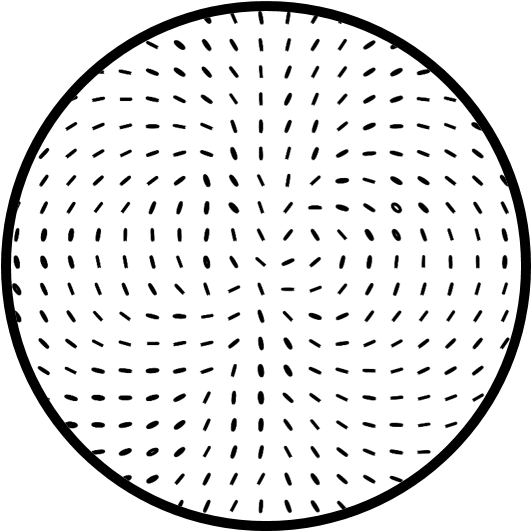


**Fig. S2**  **a** and **b** distributions of the vector field reconstructed from the experimentally measured intensity profiles shown in Fig. 3d.

**
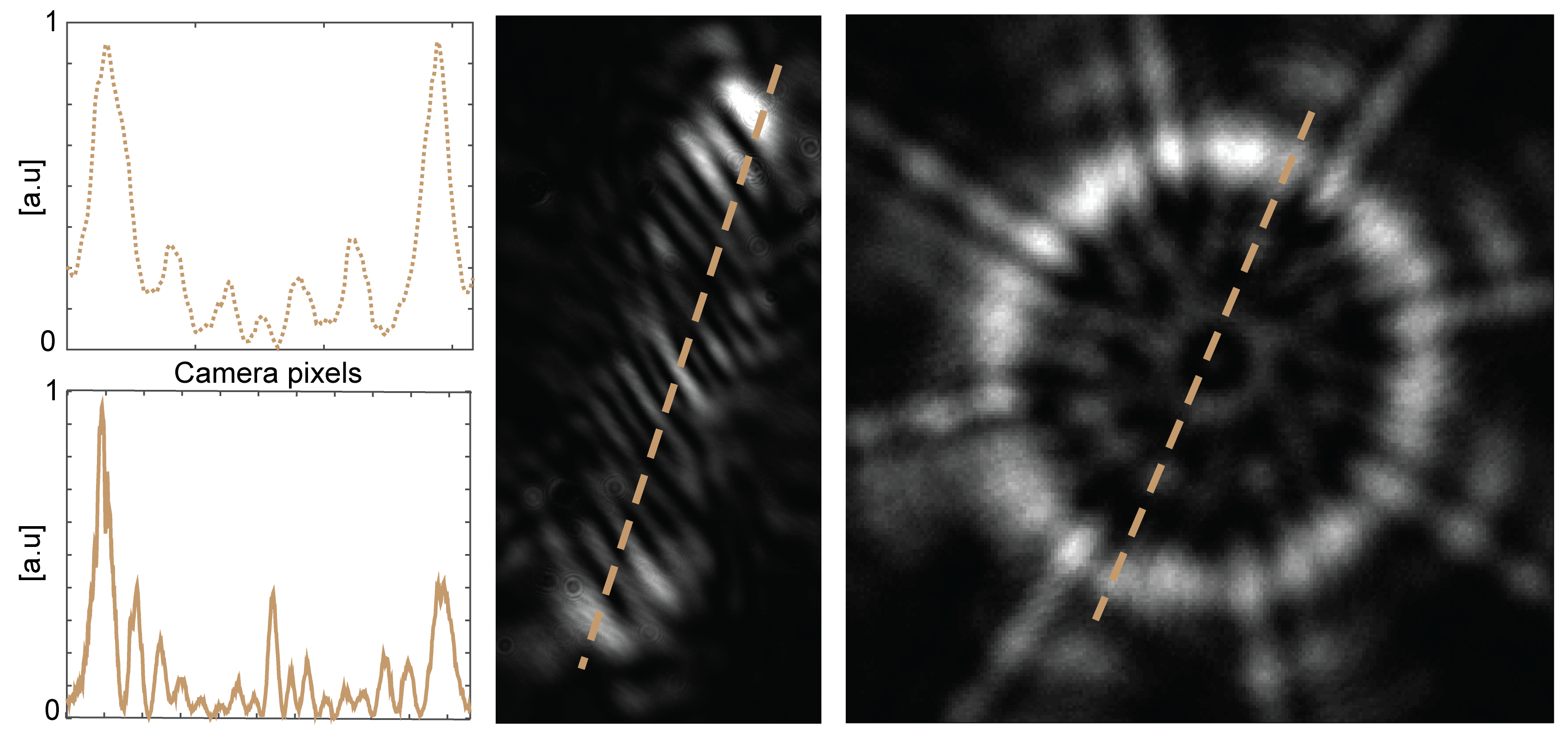
Fig. S1**  Experimental results of imaging a Gaussian beam through a SPP with m=16, using a cylindrical (left) and a spherical (right) lens.

# **Note 1: High order SPP Scan**

To examine the profile of the printed template and the conversion of the desired profile to a transparent layer we built a scanning system. The sample was scanned using a laser beam focused on the focal plane of the objective in a Nikon Ti-2 microscope. A 3D scan was performed – in NxN lateral positions, a z-stack comprising of M slices was acquired per position in a confocal-like manner (although no pinhole was used in the detection path).

Due to the highly scattering nature of the sample, while an in-focus PSF is highly concentrated, with defocus the energy quickly dissipates arbitrarily, prohibiting a detailed analysis of the out-of-focus PSF. To find the in-focus plane, each 8x8 camera pixels were binned, and a small ROI (4x4 or 6x6, post-binning) was acquired around the focused laser position. Estimation of the axial mask surface height of the mask in each z-stack position (one of NxN) was done in two steps. First, a score was given to each frame of the z-stack, corresponding to its proximity to the optimal focus. Next, the scores were fitted with a gaussian as a function of z, to find the axial position of best focus. We have found that a robust score for the focus proximity of a frame is the sum of the brightest pixels in the frame. This is susceptible to the quick energy dissipation, while maintaining robustness to the randomly aberrated shape of the PSF. The exact number of pixels (larger than 2) had little significance over the estimated profile.

The sample of the high order SPP was scanned with N = 60 (3600 lateral points), with a lateral resolution (pixel pitch) of 66 μm. Each z-stack comprised of M = 11 frames at 50 μm steps.

After obtaining the upper surface profile measurement and validating its compatibility with our design, we proceeded to measure the phase distribution resulting from the light passing through the mask. For this purpose, we utilized a holography system to reconstruct the phase distribution generated by the mask and compared it to our design (Fig. S4). The holography setup is described in supplementary Note 8.

**Fig. S3**  The scan result and a comparison to the original design of the high-order SPP (‘Pizza’ shape). Left- a heightmap of the first layer of the ‘Pizza’ SPP. Center- the corresponding reversed heightmap of the first layer, which should correspond to the template-design. Right: the heightmap of the original template-design of the ‘Pizza’ SPP.


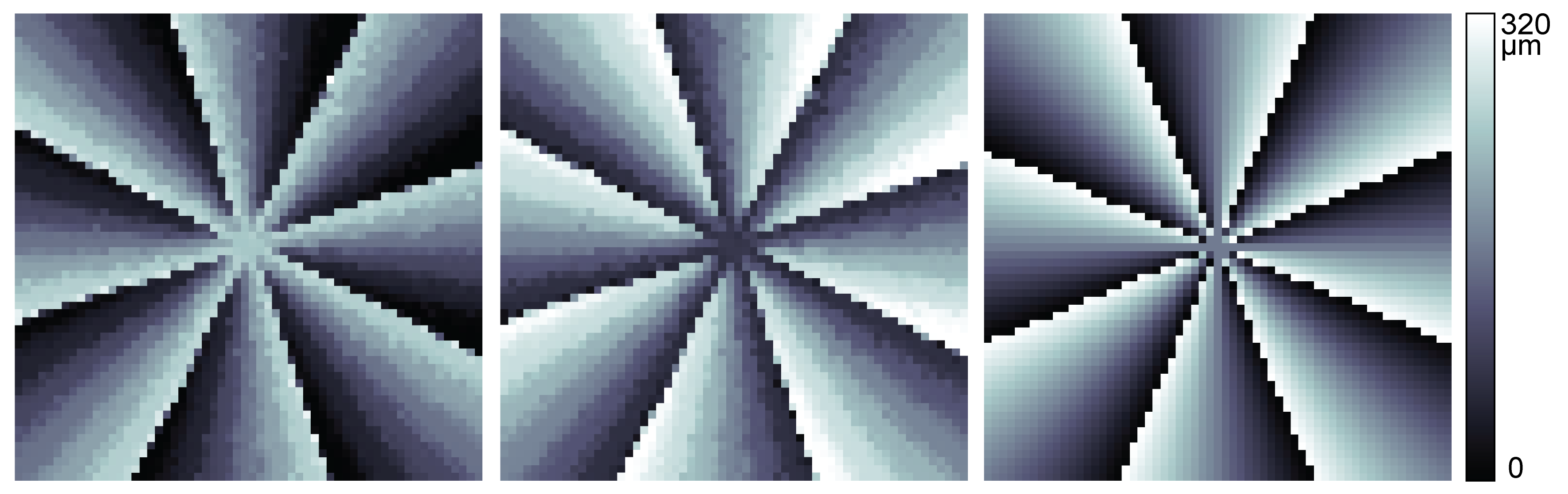


**
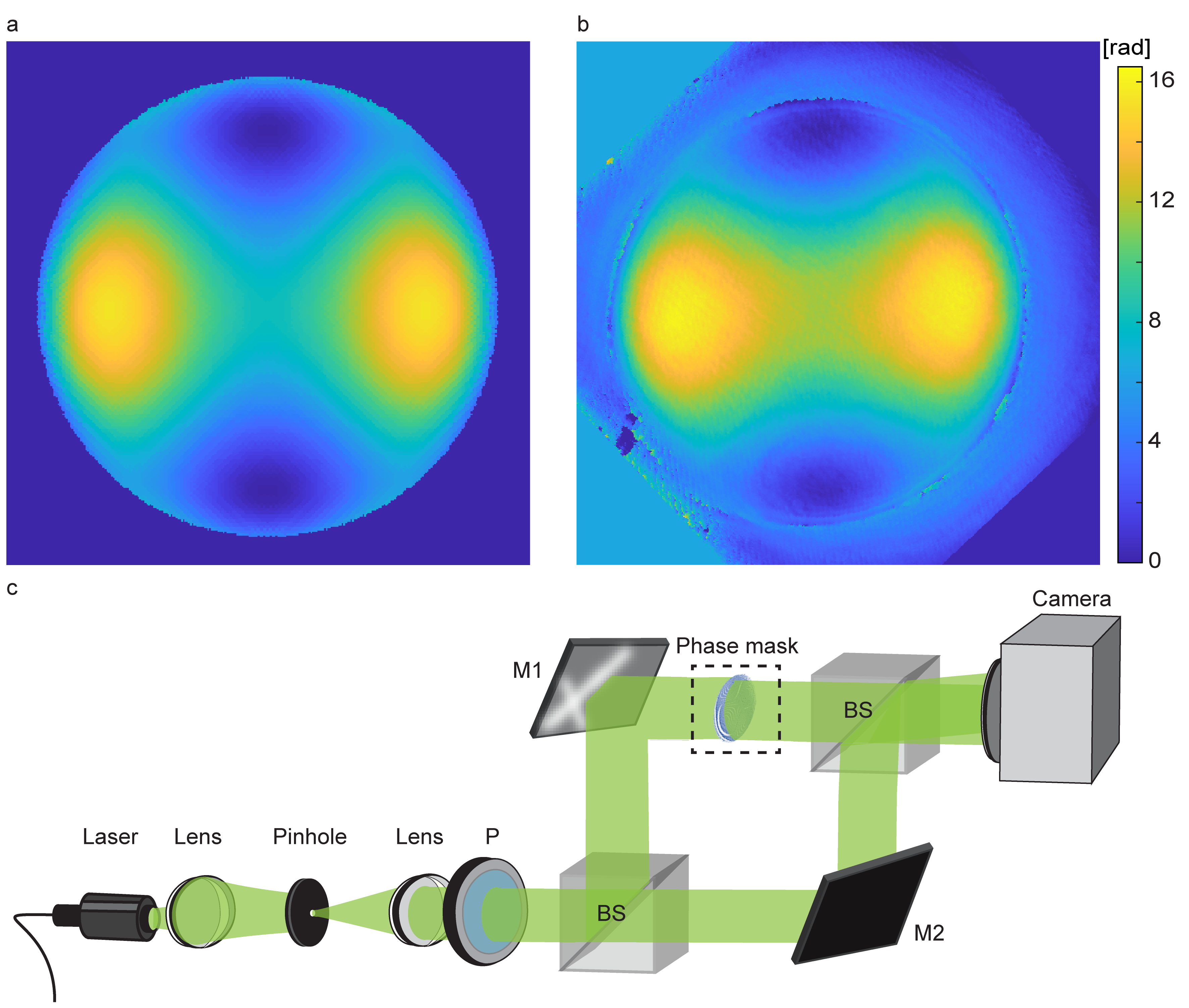
Fig. S4** Phase measurement. **a** designed phase pattern, **b** unwrapped phase map reconstruction from the holography system. **c** The holography system: P-polarizer, BS- beam splitter, M1, M2- mirrors.

# **Note 2: Surface Roughness**

Upon verifying the match between the design and the polymerized element, our next objective was to quantify the surface roughness of our template, and to ensure the roughness does not significantly hinder optical performance. We performed a fine-grained scan of the 'Pizza Vortex' template using the dynamic confocal microscope - Leica DCM3D. The data processing is performed using SensoMap Turbo software. The pixel size is $\sim3 \mu m$ (Fig. S5a).

**
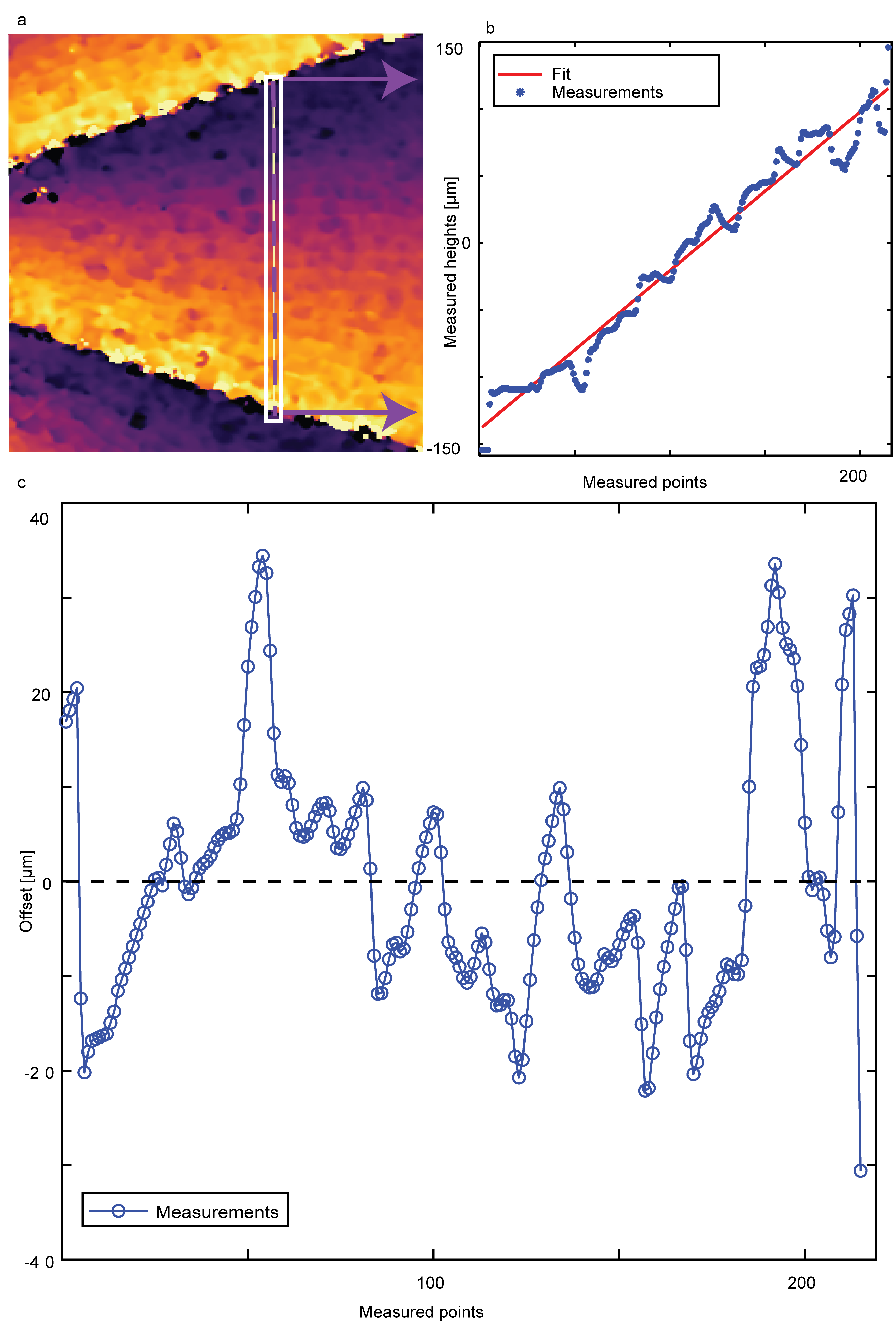
Fig. S5**  Surface roughness measurement: **a** The scanned template, – the representative line is dashed. **b** The measured points (blue) and the fitted line (red). **c** The measured points after subtracting offset (measured from the fitted line).

Next, we chose one representative slope from the measurement and calculated its linear fit (Fig. S5b), We subtracted it from the raw data obtain the roughness estimate (Fig. S5c). Finally, we calculated the roughness values:

**
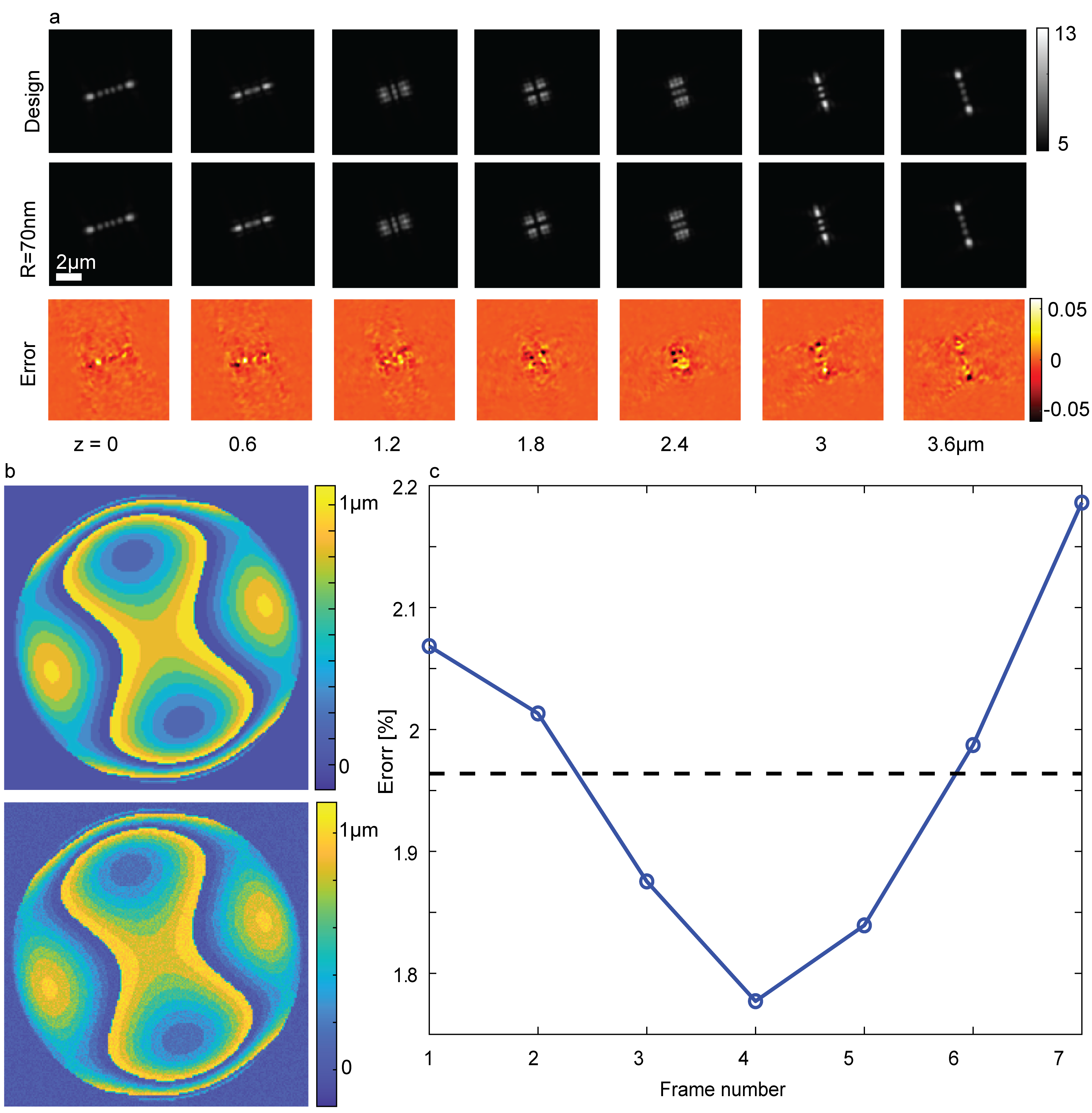
Fig. S6**  Optical performance simulation. **a** Simulated images of point emitters at different axial positions with the designed phase mask for photolithography fabrication without roughness (top), with $\pm70nm$ roughness (middle) and the calculated error (down). **b** A heightmap of the phase mask without (top) and with (bottom) roughness. **c** The total calculated error per frame.

$${\left( 1 \right) R}_{a}=\frac{1}{n}\sum_{i=1}^{n} \left| dh_{i} \right|=10.4 \mu m$$

$$\left( 2 \right) R_{q}=R_{RMS}=\sqrt{\frac{1}{n}\sum_{i=1}^{n} dh_{i}^{2}}=13.06 \mu m$$

Where n is the number of measured points and *dh* is the deviation of the measured point number (i) from the straight line.

The optical effect of the printing roughness scales exactly like the phase mask, i.e., translating this roughness to an equivalent roughness in a photolithographically fabricated mask in air, we obtain:

$$R_{air}\cdot dn_{air}=R_{\mathrm{NIM}}\cdot dn_{\mathrm{NIM}}\to R_{air}=R_{\mathrm{NIM}}\cdot\frac{dn_{\mathrm{NIM}}}{dn_{air}}=13.06\cdot\frac{0.002}{0.46}\sim\boldsymbol{57 nm}\boldsymbol{\sim}\frac{\boldsymbol{\lambda}}{\boldsymbol{10}}$$

To estimate the effect of such roughness on the DOE properties, we simulate a lithographic mask with similar roughness $(\mp70 nm)$ and calculate its the optical performance. We estimate the error of the resulting PSF due to surface roughness to be no more than 2.2% (Fig. S6).

According to the specifications provided by Lithoz, the roughness is expected to be less than 1 $\mu m$, using Alumina. Our measurements show larger roughness ($\sim10 \mu m$), which we attribute to the challenging geometry and our scanning method.

# **Note 3: Dispersion**

To calculate the dispersion of our DOE, affected by the two materials (layer 1 and layer 2), we measured the refractive index at seven different wavelengths (Anton Paar, Abbemat MW). We then fit a curve to the discrete measurements and found the offset between the two curves. After adding the constant offset to the lower curve we obtain a deviation between the curves that that does not exceed $4\cdot{10}^{-5}$, which is less than 2% of the refractive index difference (Fig. S7).

**
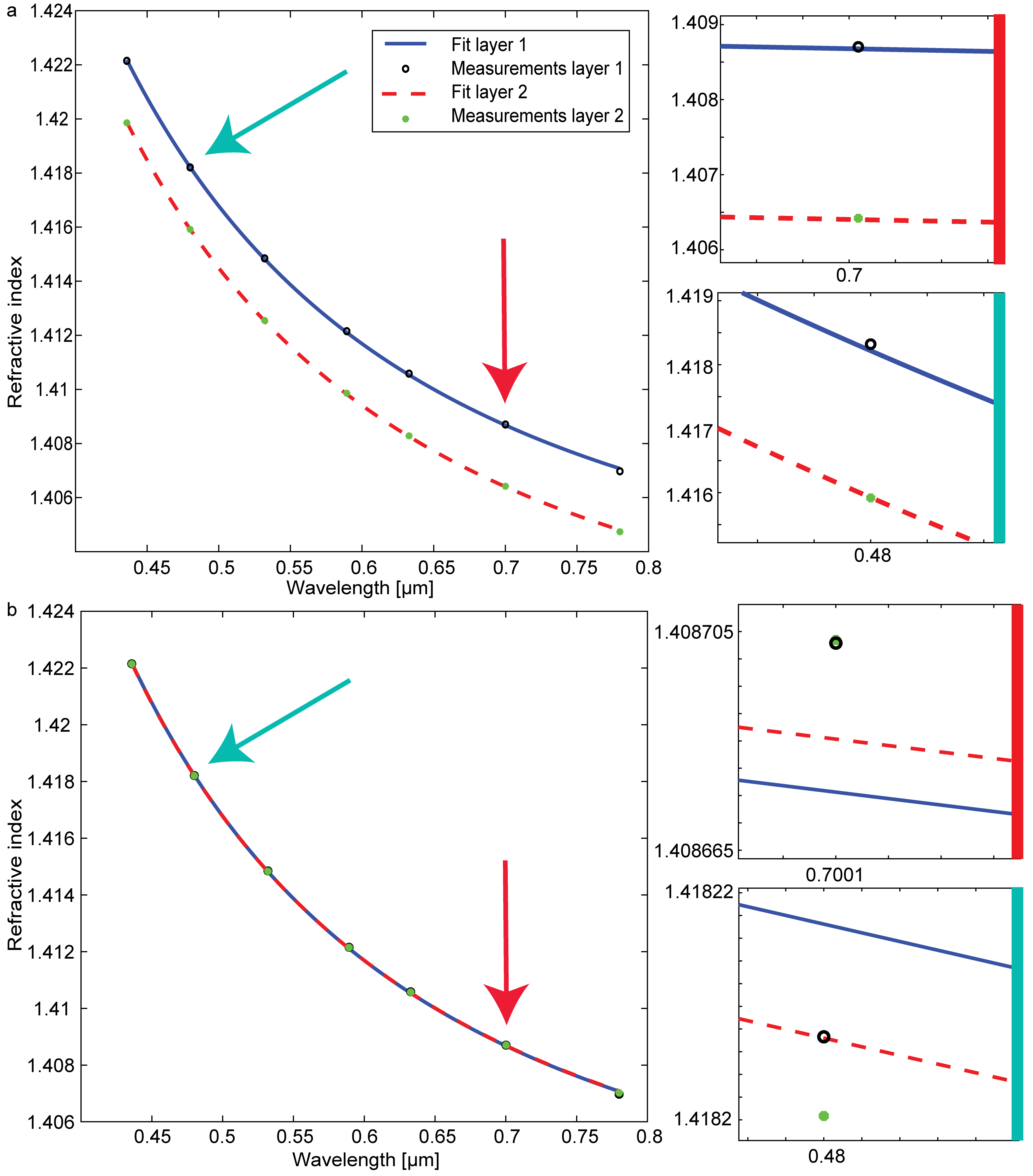
 Fig. S7**  Dispersion. **a** and **b** the dispersion curves and the measured points before and after adding a constant offset. Two zoomed areas (around 480 nm and 700.1 nm) are marked in cyan and red respectively.

# **Note 4: Stability measurement**

To assess the long-term stability of our DOE, we conducted measurements on fluorescence emitters using the same DOE (Multi TP Phase Mask) in two separate experiments, three months apart. Results show a high level of compatibility between our measurements, with slight variations observed likely attributed to a minor disparity in the positioning of the mask (Fig. S8).

**Fig. S8** Stability. Experimental images of green (515 nm) fluorescent microspheres at different axial positions imaged using same DOE in **a** February 2023 and **b** May 2023 respectively.


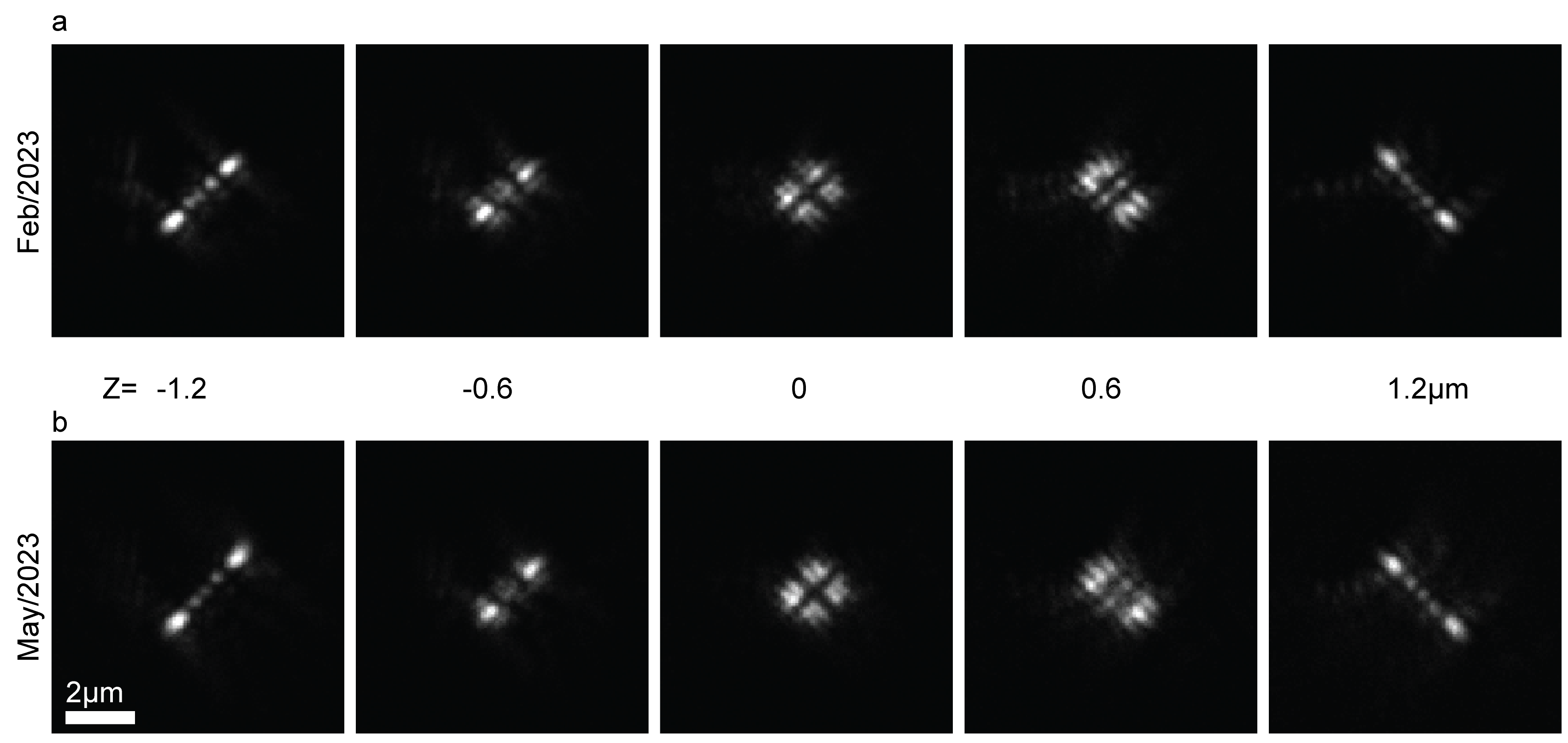


**Note 5: Performance comparison**

We conducted a performance comparison between the experimental results of the double helix and the Tetrapod multi color masks and their simulations. (Fig. S9). We show high optical performances that have a good match to the simulations. The small changes between the simulation and the experimental data have small effect on our results because the reconstructions of the phase masks for the analysis step is based on the experimental data and not on the original design of the phase masks.

**
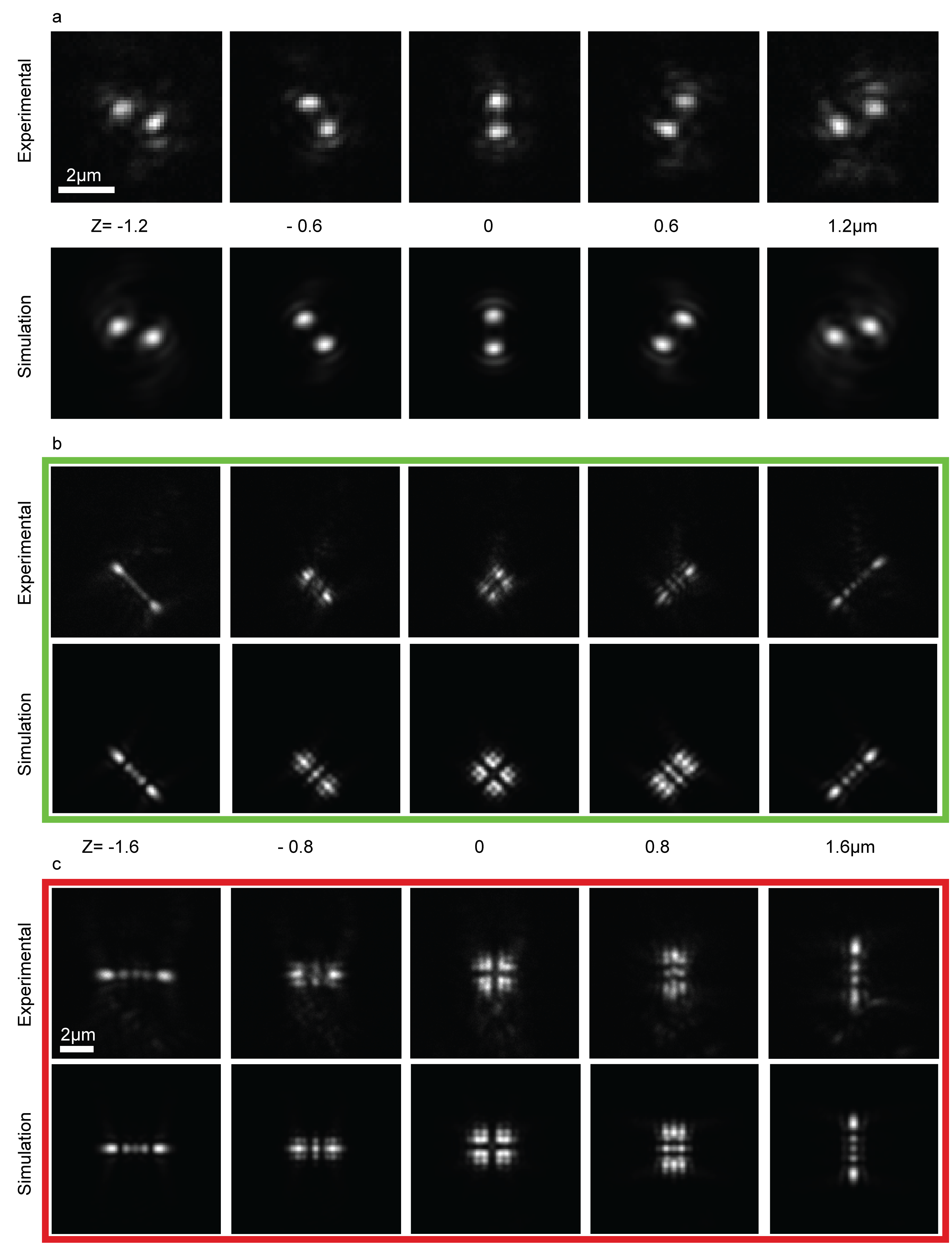
**

**Fig. S9**  Z- stack comparisons. Experimental and simulated z- stacks (images of fluorescent microspheres at different axial positions) with the double helix phase mask **a** and the Tetrapod multi-color phase masks for green 515 nm **b** and dark red 680 nm **c** fluorescent microspheres.

# **Note 6: Staircase effect**

For measuring the staircase effect, namely, quantization of the z-heights of the fabricated masks, on the functionality of our DOE, we discretized the design of the SPP and simulated its performance numerically. Fine discretization, i.e. 27 number of steps (Fig. S10 first row), exhibits total error of less than 2%, and coarse discretization, i.e. 14 steps (Fig. S10 second row) - less than 6%.

Moreover, the axial resolution of the Lithoz printer is reported to be around $25 \mu m$ (derived from layer thickness) which gives a minimal phase difference of $\Delta\phi_{\min}=2\pi\frac{1}{10}$. This value is similar to the value of discretization in photolithography fabrication, where the axial step size (derived from time of etching) is around 150 nm with RI difference of $dn\sim0.5$, yielding a minimal phase difference of $\Delta\phi_{\min}=2\pi\frac{3}{20}$.

**
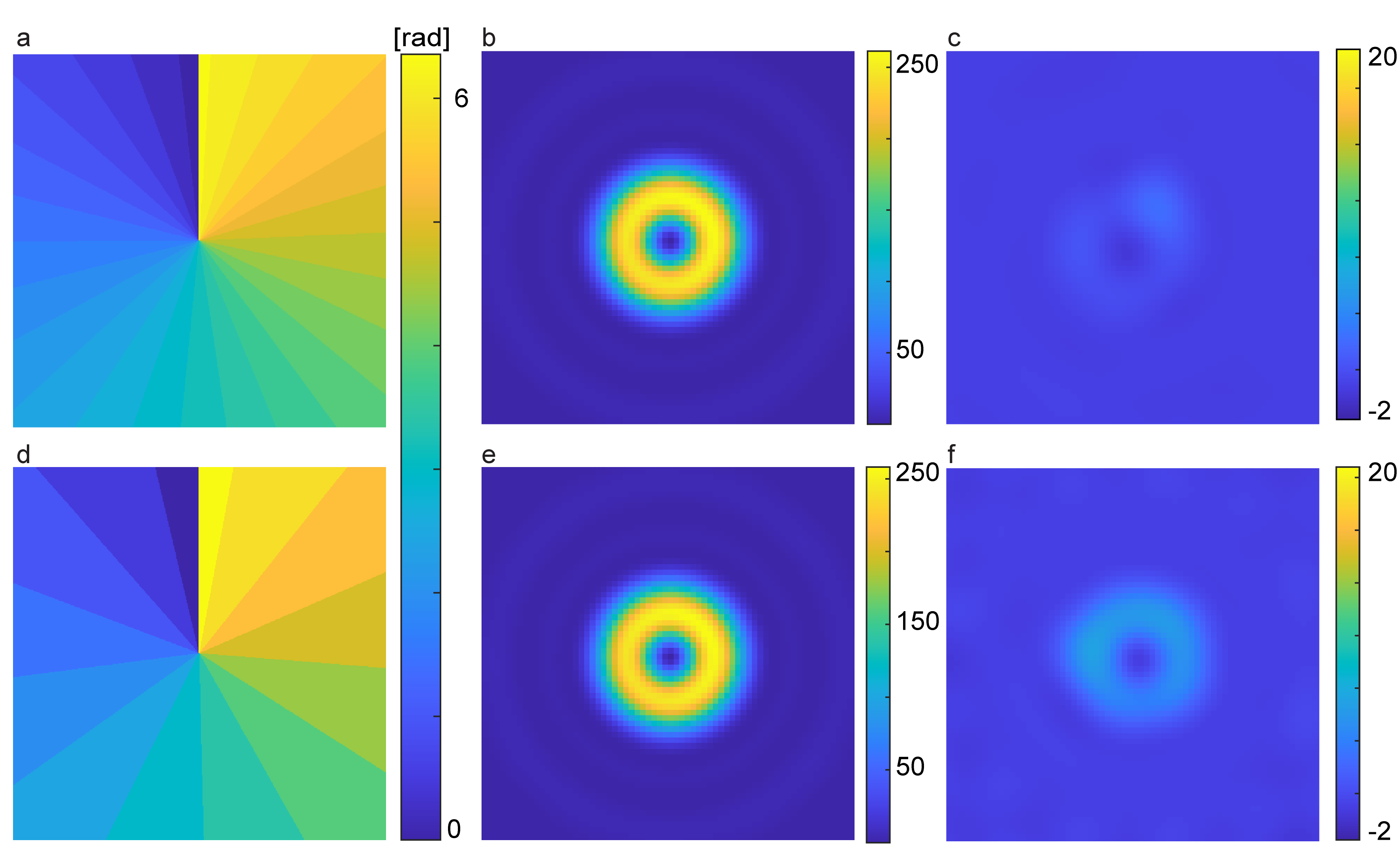
Fig. S10**  Simulations of the staircase effect. Fine and coarse discretization (first and second rows respectively) of the SPP with L=1. **a** and **d** The phase map of the SPP after discretization **b** and **e** The examined optical performance of the SPP- a ring of intensity. **c** and **f** The calculated error –subtracting the rings in **b** and **e** from the ring achieved in the optimal design.

# **Note 7: Choosing 3D printing technology and materials**

We printed our templates using two different 3D printing technologies:

1) Nanoparticle jetting-based (X-Jet)

2) photopolymerization-based printing (Lithoz, CeraFab printer)

Although both technologies performed adequately for our needs, our method is not limited to one of those 3D printing technologies. The critical parameter for choosing the 3D printing technology is the resolution of the printer. For example, CeraFab printer (Lithoz technology) have a minimal layer thickness of 20-25$\mu m$, this defines the minimal axial resolution to be 20-25$\mu m$. The lateral resolution of the Lithoz printer is defined by the pixel size (25$\mu m$) of the digital micromirror device (DMD) which produces the layered image that polymerizes the slurry in the desired pixels.

Any 3D printing technology that has similar resolutions is likely to be suitable for our fabrication method. The material of the printing template is not of high importance, because in the following steps of the fabrication method we convert the template to transparent materials and ultimately the template is not part of the DOE.

To convert the template to transparent and uniform materials we chose two materials that meet the following requirements:

1. High optical transparency
2. The difference in refractive index between the two materials should be compatible with the design
3. One of the materials should be separable from the template
4. The two material should be with good adhesion to each other but without any inter-diffusion between the two layers of the DOE.

In this work, we chose to work with two transparent silicones that gave us the following refractive index differences:

| DOE: | Designed refractive index difference | Maximum height (µm) | Wavelength (nm) |
| --- | --- | --- | --- |
| Fresnel micro-lens array | 0.002 | 320 | 640 |
| SPP L=1 | 0.002 | 257 | 514 |
| SPP L=2* | 0.004 | 257 | 514 |
| “Pizza” SPP L=8 | 0.002 | 320 | 640 |
| “Pizza” SPP L=16 (SI) * | 0.00126 | 320 | 405 |
| Double Helix | 0.0023 | 275 | 668 |
| Multi-color Tetrapod | 0.0034 | 600 | 680,515 |

*for fabricating those parts, we use the same template as one row above, and adjust to different optical properties (topological charge, wavelength) by changing the RI differences.

**Table S1.**  Design parameters of different DOEs used in this work

**Note:** In the table we describe the parameters of the initial design, in practice, sometimes it is necessary to adjust the geometry of the part or the RI differences due to imperfections in the 3D printing/RI differences.

# **Note 8: Material homogeneity**

To estimate the optical homogeneity of our materials, we built a digital-holography system, and conducted the following measurement. We placed a lithographically-fabricated Tetrapod phase mask with a known profile in the object path of the system and reconstructed the phase distribution created by the phase mask (Fig. S11a). Next, we placed silicone slabs in the object-beam path, right after the phase mask we wish to reconstruct. We prepared three different slabs, with different properties. The silicones slabs were held by high-quality glasses to ensure flatness, such that all aberrations, if present, are attributed to inhomogeneity. First, we placed a thick slab of the material used for the second layer of our DOE and we successfully reconstructed the same phase distribution (Fig. S11b), without any observable degradation of the reconstruction. Next, we used a slab consisting of two silicones layers, resembling the fabricated masks, albeit without any phase pattern inside (Fig. S11c). Holography analysis was performed as described previously^1^, based on the mathematical formulation presented in Verrier et al.^2^. Phase reconstruction was performed by grabbing an interference image (a hologram) of a beam of light passing through the phase object of interest, with a reference beam, as depicted by (Fig. S11e). In brief, the interference holds phase information, which enables a complete wavefront reconstruction at a desired plane, by means of backpropagation^1^.

**
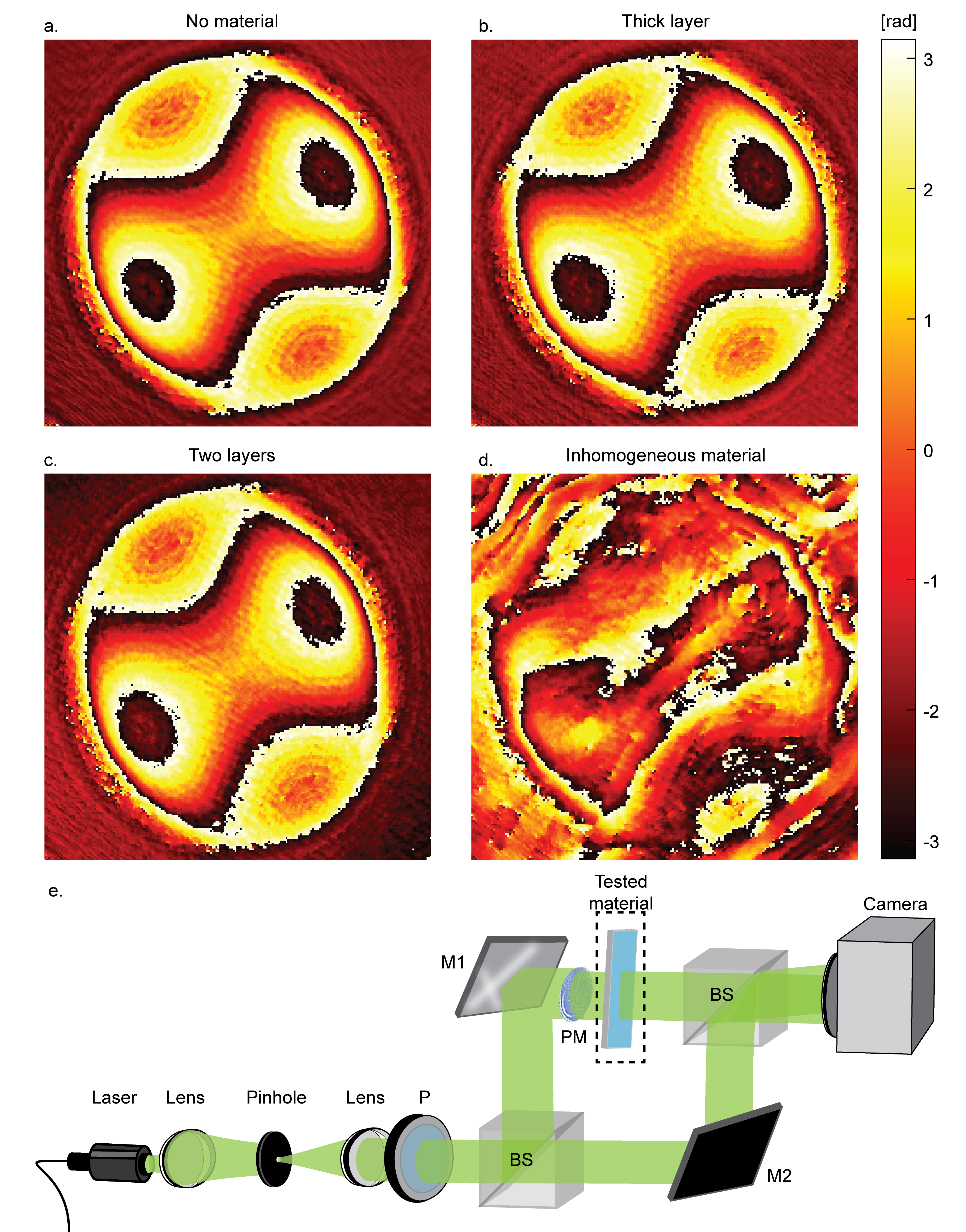
Fig. S11** Material homogeneity measurements. **a-d** The reconstructed phase distributions of the TP phase mask and blocks of different materials measured by the holography system: **a** no material **b** Thick block of the second layer material. **c** two materials one above the other of the two DOE layers. **d** Inhomogeneous material. **e** the holography system: pinhole- pinhole, P-polarizer, BS- beam splitter, M1, M2- mirrors. PM- Tetrapod phase mask.

# **Note 9: Photon efficiency**

For comparing the photon efficiency of our DOEs to a standard manufactured DOE, we utilized a simple optical setup shown in Fig. S12a. A collimated beam passed through the phase mask and was then focused by a lens onto the camera. The position of the lens was changed in order to add a quadratic phase that approximates the effect of significant defocus. For each phase mask, we acquired and averaged 10 images. To calculate the attenuation of each mask, we integrated the signal over a small area containing the main PSF. We found that our phase mask possesses similar photon efficiency as a photolithographically fabricated phase mask ($\sim$98%- integrated counts of $3.14\cdot{10}^{5}$ and $3.2\cdot{10}^{5}$, respectively for the same dashed rectangular area shown in Fig. S12b,c).

**_
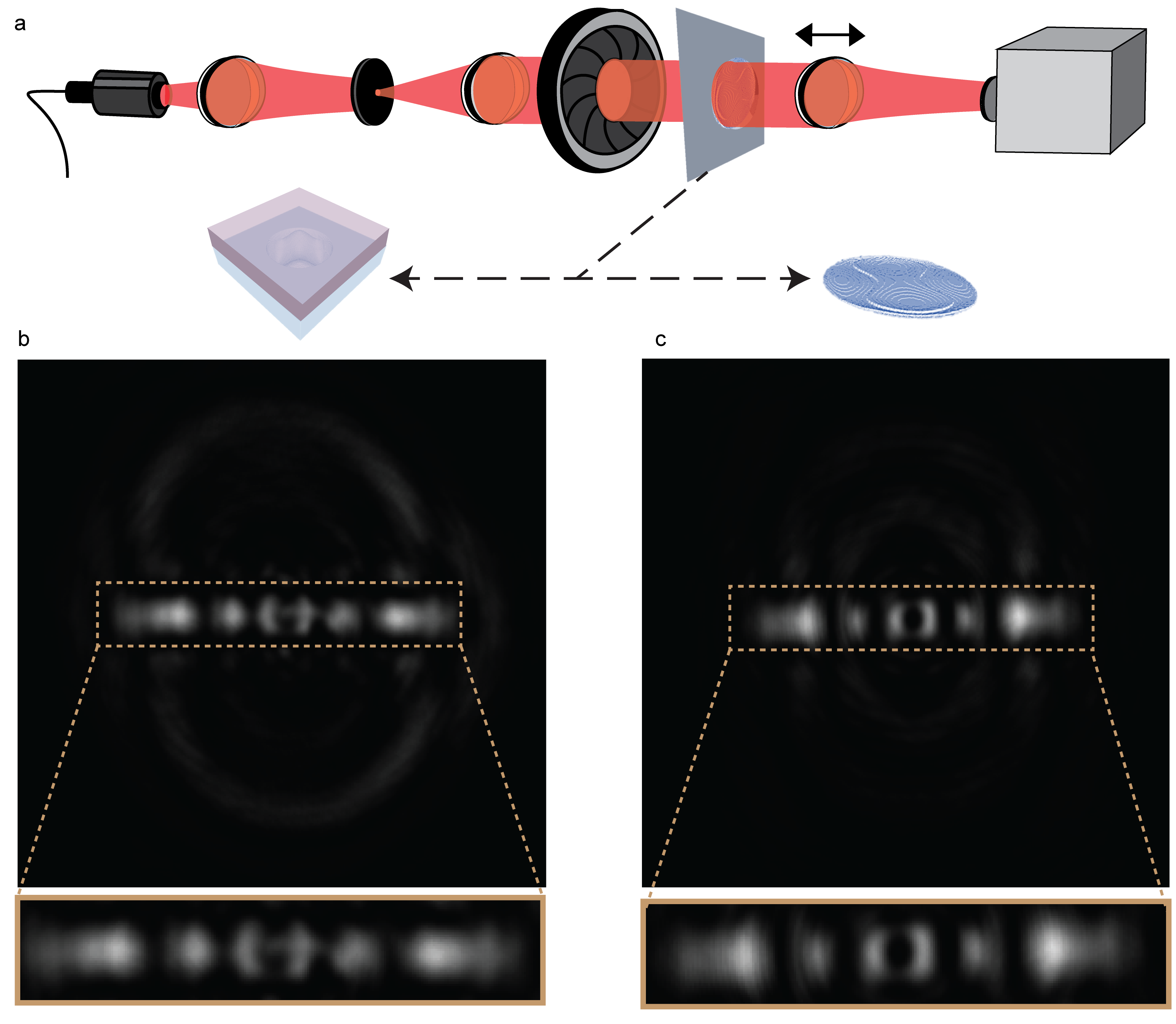
_**

**Fig. S12** Photon efficiency. **a** The optical system for the photon efficiency measurement: a collimated beam passing through the tested phase mask and focus by a lens on the camera. **b** and **c** The images acquired by the camera after placing our phase mask and photolithographically fabricated phase mask, respectively, in the optical system mentioned in **a**. Dashed rectangular- the region extracted from the image, used for photon efficiency calculation.

**Fig. S12** . Photon efficiency . (a) The optical system for the photon efficiency measurement: a collimated beam passing through the phase mask and focus by a lens on the camera. (b) and (c) The images acquired by the camera after placing our phase mask and photolithographically fabricated phase mask in the optical system mentioned in a. Dashed rectangular- the region extracted from the original image used for photon efficiency calculation.

**_Fig. S12_** _. Photon efficiency . (a) The optical system for the photon efficiency measurement: a collimated beam passing through the phase mask and focus by a lens on the camera. (b) and (c) The images acquired by the camera after placing our phase mask and photolithographically fabricated phase mask in the optical system mentioned in a. Dashed rectangular- the region extracted from the original image used for photon efficiency calculation._

**_Fig. S12_** _. Photon efficiency . (a) The optical system for the photon efficiency measurement: a collimated beam passing through the phase mask and focus by a lens on the camera. (b) and (c) The images acquired by the camera after placing our phase mask and photolithographically fabricated phase mask in the optical system mentioned in a. Dashed rectangular- the region extracted from the original image used for photon efficiency calculation._

# **Note 10: Experimental PAINT performance comparison**

In the main text, Fig. 5 presents PAINT imaging results achieved using our double-helix DOE. To assess the performance of our DOE, we compare these results to those obtained in a previous experiment conducted under similar conditions but employing a commercial DOE ^3^ .  Fig. S13a provides a qualitative comparison of the raw data frames from the experiments. In the lower portion of Fig. S13, we quantitatively compare the localization precision and photon counts. Our experiment achieved similar (even slightly improved) resolution to previous work (13.5 nm, 22 nm compared to 14.5 nm, 30.7 nm).

**_
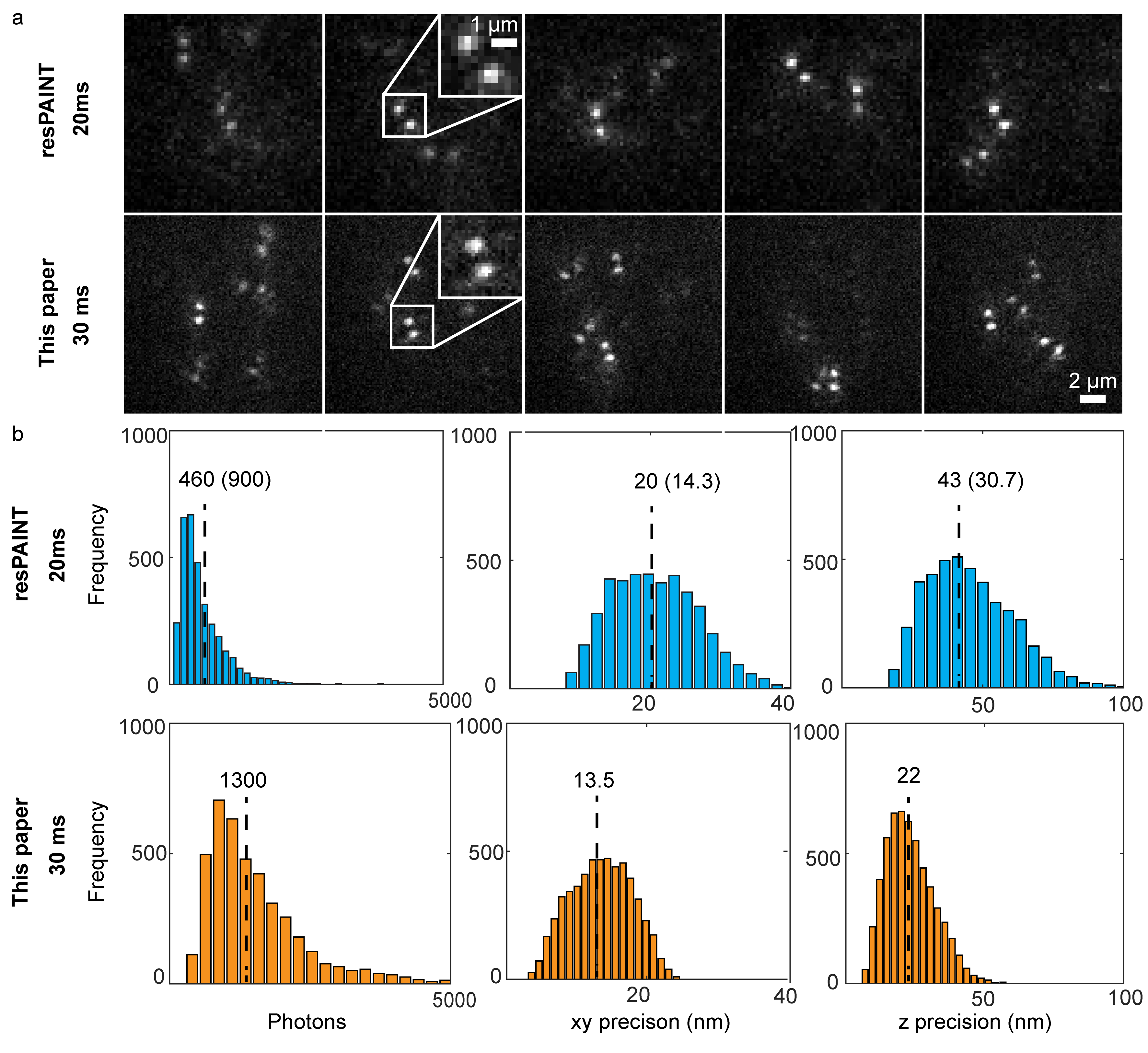
_ Fig. S13** Comparison between additive manufacturing and commercial phase mask optical performance. **a** Representative raw DHPSF frames for commercial phase mask (resPAINT) and this paper. Contrast has been adjusted for comparison. **b** Histograms of intensity, xy- and z-precision for a commercial phase mask used for resPAINT^3^ and for the mask prepared in this work. Dotted lines report median values. The parenthesis is the exposure time and power density adjusted equivalent for direct comparison

# **Note 11: Movie details**

Supplementary movie 1: **Super-resolution reconstruction of mitochondria**. 3D rendering of mitochondria spanning a 4 μm axial range (colormap 0-4 μm), corresponding to main text Fig. [6g](https://www.nature.com/articles/s41592-020-0853-5#Fig3). Scale bar = 5 μm.

**References**

1. Orange-Kedem, R. *et al.* 3D printable diffractive optical elements by liquid immersion. *Nat Commun* **12**, 1–6 (2021).

2. Verrier, N. & Atlan, M. Off-axis digital hologram reconstruction: some practical considerations. *Appl Opt* **50**, H136 (2011).

3. Sanders, E. W. *et al.* resPAINT: Accelerating Volumetric Super‐Resolution Localisation Microscopy by Active Control of Probe Emission. *Angewandte Chemie* **134**, 1–8 (2022).
